# Precision Medicine in Lung Cancer Screening: A Paradigm Shift in Early Detection—Precision Screening for Lung Cancer

**DOI:** 10.3390/diagnostics15121562

**Published:** 2025-06-19

**Authors:** Hsin-Hung Chen, Yun-Ju Wu, Fu-Zong Wu

**Affiliations:** 1 Department of Medical Education and Research, Kaohsiung Veterans General Hospital, Kaohsiung 813414, Taiwan; derekchen@vghks.gov.tw; 2 Department of Radiology, Kaohsiung Veterans General Hospital, Kaohsiung 813414, Taiwan; yjwu@vghks.gov.tw; 3 Faculty of Medicine, School of Medicine, National Yang Ming Chiao Tung University, Taipei 112, Taiwan; 4 Faculty of Clinical Medicine, National Yang Ming Chiao Tung University, Taipei 112, Taiwan

**Keywords:** lung cancer screening, precision medicine, prediction model

## Abstract

Lung cancer remains the leading cause of cancer-related mortality globally, largely due to late-stage diagnoses. While low-dose computed tomography (LDCT) has improved early detection and reduced mortality in high-risk populations, traditional screening strategies often adopt a one-size-fits-all approach based primarily on age and smoking history. This can lead to limitations, such as overdiagnosis, false positives, and the underrepresentation of non-smokers, which are especially prevalent in Asian populations. Precision medicine offers a transformative solution by tailoring screening protocols to individual risk profiles through the integration of clinical, genetic, environmental, and radiological data. Emerging tools, such as risk prediction models, radiomics, artificial intelligence (AI), and liquid biopsies, enhance the accuracy of screening, allowing for the identification of high-risk individuals who may not meet conventional criteria. Polygenic risk scores (PRSs) and molecular biomarkers further refine stratification, enabling more personalized and effective screening intervals. Incorporating these innovations into clinical workflows, alongside shared decision-making (SDM) and robust data infrastructure, represents a paradigm shift in lung cancer prevention. However, implementation must also address challenges related to health equity, algorithmic bias, and system integration. As precision medicine continues to evolve, it holds the promise of optimizing early detection, minimizing harm, and extending the benefits of lung cancer screening to broader and more diverse populations. This review explores the current landscape and future directions of precision medicine in lung cancer screening, emphasizing the need for interdisciplinary collaboration and population-specific strategies to realize its full potential in reducing the global burden of lung cancer.

## 1. Introduction

Lung cancer remains the leading cause of cancer-related mortality worldwide. Despite advances in treatment, the prognosis of lung cancer largely depends on the stage at diagnosis, with early detection offering the best chance for curative intervention. Traditional lung cancer screening programs—typically using low-dose computed tomography (LDCT)—have demonstrated a significant reduction in mortality by identifying cancers at an earlier, more treatable stage [1]. However, these programs are often applied in a “one-size-fits-all” manner, which can lead to overdiagnosis, unnecessary biopsies, and false positives, particularly in populations not well-represented in initial screening trials [2,3]. Clinical trials and meta-analyses have confirmed that lung cancer screening among high-risk smokers effectively reduces lung cancer mortality [4,5]. However, in Asia, non-smoking populations—particularly women—may still be at high risk for lung cancer due to genetic, environmental, and other region-specific factors [6,7,8]. The high prevalence of subsolid nodules (SSNs) in this group increases the risk of overdiagnosis and overtreatment [9]. Therefore, precision and personalized approaches to lung cancer screening are especially critical for non-smoking Asian populations to improve outcomes and minimize harm [10]. To address these challenges, precision medicine has emerged as a transformative approach, aiming to tailor lung screening strategies to individual risk profiles and biological characteristics.

## 2. Understanding Precision Medicine

Precision medicine represents a transformative approach to healthcare that emphasizes the personalization of medical decisions, interventions, and treatments based on an individual’s unique genetic profile, environmental influences, and lifestyle behaviors [11]. Rather than applying uniform protocols to all patients, precision medicine seeks to tailor healthcare strategies to the specific biological and contextual characteristics of each person, thereby maximizing effectiveness and minimizing unnecessary interventions. Within the realm of lung cancer screening, this personalized model plays a critical role in refining and optimizing screening practices [12,13,14]. It involves the integration of diverse and multidimensional data inputs, including demographic and clinical risk indicators, such as a patient’s age, smoking habits, and family history of cancer. Additionally, it leverages insights from genetic and epigenetic markers that may reveal inherited susceptibilities or molecular alterations associated with cancer development [15]. Advanced imaging techniques also contribute radiomic features—quantitative descriptors derived from CT or LDCT scans—that can capture tumor heterogeneity and subtle patterns indicative of malignancy [16,17,18]. Environmental and occupational exposure histories further enrich the risk profile by accounting for external carcinogenic influences [19,20]. Moreover, biomarkers extracted from non-invasive samples, such as blood or exhaled breath, offer dynamic measures of biological activity that may signal early disease processes [21,22,23]. By synthesizing this wide array of individualized data, precision medicine enables more accurate risk stratification, facilitates early detection of high-risk individuals, enhances the overall effectiveness of lung cancer screening programs, and significantly reduces the physical, psychological, and economic harms often associated with population-wide or indiscriminate screening efforts [24].

## 3. Traditional Lung Cancer Screening: Challenges Posed by Inconsistent Screening Guidelines

Conventional LDCT screening guidelines primarily emphasize age and smoking history [1,25,26]. For example, the United States Preventive Services Task Force (USPSTF) recommends annual LDCT for adults aged 50–80 years with a 20 pack-year smoking history who currently smoke or have quit within the past 15 years [26]. While this strategy has proven effective in reducing lung cancer mortality, it presents several limitations. Notably, it underrepresents non-smokers, despite a substantial proportion of lung cancer cases—particularly in Asian populations—arising in individuals with no history of smoking, thereby excluding them from early detection programs [27,28]. Additionally, overdiagnosis remains a concern, as some detected lesions, such as subsolid or indolent adenocarcinomas, may never progress to clinically significant disease, leading to unnecessary treatments and psychological burden [29,30,31]. The high rate of SSNs in Asian populations also contributes to increased patient anxiety, unwarranted invasive procedures, and rising healthcare costs [9]. These challenges highlight the urgent need for a more refined, individualized screening approach—one that precision medicine is particularly well-suited to support.

Lung cancer risk factors in Asian populations differ significantly from those in heavy-smoking Western populations, highlighting the importance of adopting distinct screening criteria [28]. However, variations in eligibility rates, efficiency ratios (ERs), inclusion rates, and the proportion of detected lung cancers across different screening strategies present a critical challenge. Developing precise, tailored screening criteria is essential to effectively identify high-risk individuals and enhance the overall impact of lung cancer screening in Asian populations [13]. Tang et al. conducted a study to assess the efficiency of four lung cancer screening (LCS) eligible criteria according to different guidelines in a Chinese population [28]. Their study analyzed data from 31,394 asymptomatic individuals screened with LDCT between 2005 and 2022. The four guidelines assessed were the China guideline for the screening and early detection of lung cancer (CGSL), the National Comprehensive Cancer Network (NCCN), the USPSTF, and the International Early Lung Cancer Action Program (I-ELCAP) [26,32].

Among the screened individuals, 298 cases of lung cancer were diagnosed (155 women and 143 men). The eligibility rates for each guideline were 13.92% (CGSL), 6.97% (NCCN), 6.81% (USPSTF), and 53.46% (I-ELCAP). The ERs for those deemed eligible were 1.46% (CGSL), 1.64% (NCCN), 1.51% (USPSTF), and 1.13% (I-ELCAP), while the inclusion rates were 19.0%, 9.5%, 9.3%, and 73.0%, respectively. The proportions of lung cancers detected among those meeting the criteria were 29.2% (CGSL), 16.4% (NCCN), 14.8% (USPSTF), and 86.6% (I-ELCAP), as shown in Table 1. CGSL, NCCN, and USPSTF had the highest underdiagnosis in the 45–49 age group (17.4%), while I-ELCAP showed the highest missed diagnosis rate (3.0%) in the 35–39 age group. Gender differences in eligibility were significant across all guidelines (*p* < 0.001). CGSL had stricter age and smoking criteria, leading to lower inclusion rates and higher underdiagnosis rates. The study highlighted the variations in efficiency among LCS guidelines in the Chinese population. The I-ELCAP guideline had the highest eligibility rate, meaning it captured the most potential lung cancer cases, but it had the lowest efficiency ratio, indicating a higher inclusion of individuals with lower risk. Conversely, the NCCN guideline had the highest efficiency ratio, suggesting it is more selective in identifying high-risk individuals. Different screening eligibility guidelines have varying characteristics, advantages, and limitations. Therefore, precision lung cancer screening must tailor the choice of screening guideline standards based on the specific country, ethnicity, and regional context to optimize overall screening efficiency and lung cancer detection rates.

**Figure 1:**
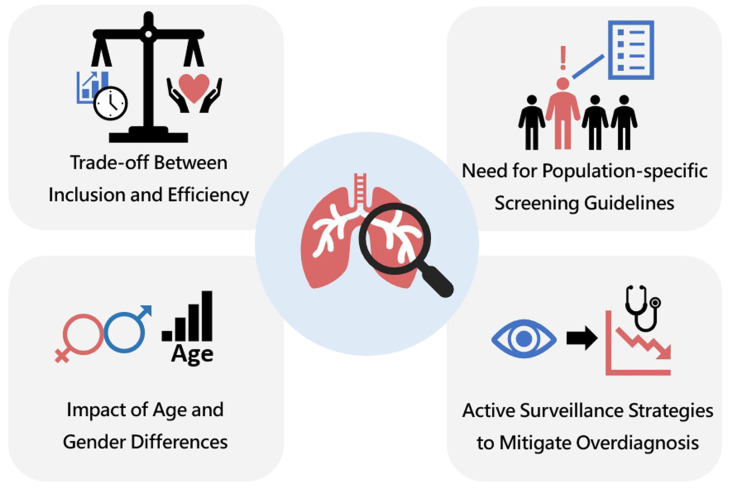
Four strategies to optimize the benefits of lung cancer screening and identify high-risk lung cancer in Asian populations. This figure illustrates four key strategies to optimize lung cancer screening and identify high-risk lung cancer cases in Asian populations. At the center is a depiction of lungs with a magnifying glass, symbolizing focused screening. The top left quadrant highlights the trade-off between inclusion and efficiency, emphasizing the need to balance broad access with resource utilization. The top right stresses the need for population-specific screening guidelines, acknowledging the unique risk profiles of Asian populations. The bottom left addresses the impact of age and gender differences, suggesting tailored approaches for more effective screening. Finally, the bottom right underscores the importance of active surveillance strategies to mitigate overdiagnosis, aiming to reduce unnecessary treatments. Together, these strategies advocate for a more precise, equitable, and effective lung cancer screening framework tailored to Asian populations.

The study provided four strategies and recommendations to optimize the benefits of lung cancer screening and identify high-risk lung cancer in Asian populations, as shown in Figure 1:

(1) **Trade-off Between Inclusion and Efficiency.**

The study highlighted the balance between eligibility rates and ERs among different guidelines. While I-ELCAP captured the most lung cancer cases, its lower efficiency ratio suggested it included a larger proportion of low-risk individuals. Conversely, NCCN had the highest efficiency ratio, meaning it was more selective but also had lower inclusion rates. This trade-off is crucial for optimizing screening strategies in different populations.

(2) **Impact of Age and Gender Differences.**

The study revealed significant underdiagnosis in younger populations (especially ages 45–49) for CGSL, NCCN, and USPSTF and a missed diagnosis rate for I-ELCAP in the 35–39 age group. Additionally, the significant gender differences in eligibility across all guidelines (*p* < 0.001) suggested the need to refine criteria to ensure equitable screening access, particularly for women who may develop lung cancer despite not meeting traditional smoking-based criteria.

(3) **Need for Population-Specific Screening Guidelines.**

The study underscored the limitations of applying Western-based guidelines (NCCN, USPSTF, I-ELCAP) directly to a Chinese population. The CGSL guideline, while designed for China, still exhibited strict criteria that may lead to underdiagnosis. This suggested that a more tailored risk-stratification approach, possibly integrating non-smoking-related risk factors, is needed to optimize lung cancer screening in Chinese and other Asian populations [13,33].

(4) **Active Surveillance Strategies to Mitigate Overdiagnosis.**

Recent discussions on lung cancer screening in Asia have highlighted concerns regarding overdiagnosis, particularly among non-smoking individuals, especially women, leading to potential overtreatment [31,34,35]. The study primarily evaluated the impact of different screening guideline criteria on screening efficiency. Given that most lung cancers detected in non-smokers in Asia belong to the adenocarcinoma spectrum, which exhibits varying growth rates, overly aggressive screening may contribute to overdiagnosis. Conversely, delayed follow-up could result in missed diagnoses, adversely affecting survival outcomes [36]. Therefore, a key clinical challenge in lung cancer screening is to refine high-risk screening criteria to identify rapidly growing, high-risk lung cancers rather than indiscriminately capturing both indolent and aggressive tumors [37]. Striking the right balance between timely detection and avoiding unnecessary interventions will be crucial in optimizing lung cancer screening strategies. Future lung cancer screening guidelines should aim to identify high-risk lung cancer patients by incorporating active surveillance to monitor interval tumor growth in ground-glass nodules and assess prognosis [38,39]. Detecting changes in tumor progression over time will be key to recognizing patients at risk of disease deterioration, allowing for timely intervention and improved clinical outcomes.

## 4. Key Elements for Effective Precision Lung Cancer Screening

Key elements for effective precision lung cancer screening include (1) risk prediction models, (2) radiomics and artificial intelligence (AI), (3) molecular and biomarker-based screening, (4) genomic profiling and polygenic risk scores (PRSs), and (5) government policy and human behavioral and environmental data integration. Several recent genomic studies have identified susceptibility loci associated with lung cancer in the Asian non-smoking population, and PRSs—by combining information from multiple single-nucleotide polymorphisms (SNPs)—can estimate an individual’s inherited risk [40,41]. Although still under development, PRSs may eventually guide screening intervals and initiation for genetically predisposed individuals, even those without a history of smoking [42,43]. In addition to these scientific advances, precision lung cancer screening must also take into account a broad range of factors, including government policies, individual health literacy, hospital management strategies, personalized lung cancer risk profiles, and clinical decision-making models [35,44]. In recent years, multiomics approaches—integrating clinical, imaging, and genomic data—have increasingly been used to develop AI-assisted personalized risk prediction models and clinical decision-support tools to accelerate the implementation of lung cancer screening in clinical practice [45,46]. These innovations are expected to enhance the effectiveness of screening programs and reduce the burden on medical personnel resulting from large-scale implementation. However, recent studies have also noted potential challenges: AI-assisted image interpretation may lead to an increased detection of non-specific pulmonary nodules, thereby raising false-positive rates [47,48]. This may also result in public misunderstanding, overdiagnosis, and overtreatment, ultimately undermining the overall benefits of lung cancer screening [49].

(1) **Risk Prediction Models.**

Recent studies have provided further evidence that risk-based models outperform traditional categorical criteria (such as age and smoking history alone) in determining eligibility for lung cancer screening, thereby improving screening efficiency [50,51]. The SUMMIT trial incorporated a multi-cancer early detection (MCED) blood test to develop a lung cancer risk prediction model, demonstrating high accuracy in risk assessment [52]. At 12 months, the LDCT screening protocol used in the trial achieved an episode sensitivity of 97.0% (95% CI: 95.0–99.1; detecting 261 of 269 lung cancer cases) and a specificity of 95.2% (95% CI: 94.8–95.6; 11,905 of 12,504 participants), with a corresponding false-positive rate of 4.8% (95% CI: 4.4–5.2). A recent study found that eight lung cancer prediction models performed inconsistently across clinical settings, underscoring the need for tailored or retrained models for accurate application [53]. Notably, all models performed poorly on biopsied nodules, likely because these nodules were already highly suspected of malignancy, making it difficult for the models to further stratify risk. This highlights the importance of developing or adapting models for specific clinical contexts. A one-size-fits-all approach is challenging, further underscoring the need to tailor models for individual clinical scenarios.

In recent years, lung cancer screening among non-smokers has gained increasing attention globally, particularly in Asia, where the prevalence of lung adenocarcinoma is high among non-smoking populations [9,54,55]. As a result, countries such as Taiwan, China, South Korea, and Japan have initiated lung cancer screening trials specifically targeting non-smokers [55]. Taiwan has gone a step further by launching a national lung cancer screening program that includes both smoking and non-smoking high-risk groups, aligning with the “Healthy Taiwan” top ten strategic initiatives. To improve the effectiveness of lung cancer screening, distinct risk prediction models and clinical decision-making frameworks should be developed for different high-risk populations [56]. These models must be tailored to specific screening contexts and adapted to the varying levels of health literacy regarding lung cancer across different population groups. Policy and implementation strategies should be responsive to these differences in awareness and attitudes. For high-risk male smokers, efforts should focus on enhancing recruitment strategies to increase screening coverage. For high-risk non-smoking Asian women, initiatives should prioritize education around screening benefits and risks, health literacy related to overdiagnosis, and shared decision-making (SDM) to reduce the potential harms of overdiagnosis and overtreatment [13]. Ultimately, applying precision lung cancer prediction models should align with the “4Ps” of modern healthcare—prevention, prediction, personalization, and participation—to effectively implement screening strategies in clinical practice and maximize public health outcomes [57].

(2) **Radiomics and AI.**

Radiomics involves extracting quantitative features from medical imaging (e.g., LDCT) to identify patterns that may not be visible to the human eye [16]. Recent studies underscore the expanding role of radiomics in improving early detection and prognosis prediction of lung cancer [38,58,59]. By extracting high-dimensional features from medical imaging, radiomics supports more informed clinical decisions. Its primary applications include distinguishing benign from malignant pulmonary nodules, evaluating the invasiveness of lung adenocarcinomas, identifying histopathologic subtypes, predicting interval growth in SSNs, predicting genetic biomarkers in EGFR mutation, and contributing to prognostic modeling for lung cancer risk assessment [60]. Overall, radiomics shows strong potential to enhance diagnostic accuracy and support personalized risk stratification in lung cancer screening. When integrated with effective patient–physician communication and SDM, it can greatly increase the precision and impact of early lung cancer detection efforts.

Numerous studies have highlighted the clinical potential of radiomics in lung cancer screening, particularly in differentiating benign from malignant pulmonary nodules and in assessing tumor invasiveness [61,62,63,64]. Research has demonstrated that radiomic signatures can distinguish malignant from benign nodules with sensitivity ranging from 76.2% to 92.85% and specificity ranging from 72.73% to 96.1%, indicating moderate to high diagnostic performance [16]. Ground-glass nodules (GGNs), which are commonly detected in lung cancer screening using LDCT in Asian populations, have been the focus of several studies demonstrating fair to good diagnostic accuracy in predicting invasive pulmonary adenocarcinoma (IPA) [65,66,67,68,69]. In research targeting SSNs, Wu et al. proposed a simplified radiomic model that integrates a nomogram based on GLCM-derived features—specifically GLCM_Entropy_log10. This model showed promising performance in distinguishing IPA from pre-invasive lesions, with a sensitivity of 84.8% and a specificity of 79.2% [70]. In a study of 1951 NLST participants, a serial longitudinal radiomics-based RRL model (S-RRL) outperformed baseline and clinical models, achieving an AUC of 0.88 and enhancing lung cancer risk stratification (NRI up to 0.29) [71]. However, a major current challenge lies in the lack of standardization across radiomics platforms developed by different vendors [72]. Establishing a unified and generalizable diagnostic model remains a crucial issue for the broader clinical implementation of radiomics in lung cancer screening.

(3) **Molecular and Biomarker-Based Screening.**

Biomarkers may improve high-risk participant selection before screening and help avoid overdiagnosis or unnecessary follow-up for suspicious or indeterminate findings during LCS, optimizing patient management and reducing harm from unneeded procedures or misclassification [73]. Traditional biomarkers, such as Cytokeratin 19 fragment (CYFRA 21-1) and carcinoembryonic antigen (CEA), are commonly used in lung cancer screening. While these markers show relatively high sensitivity for detecting malignancy, especially in advanced stages, their specificity is limited, making them less effective for early detection or distinguishing benign from malignant nodules [23]. Blood-based genetic biomarkers and liquid biopsies are promising tools in precision screening. Circulating tumor DNA (ctDNA), microRNAs, autoantibodies, and protein signatures are being studied as non-invasive methods for detecting lung cancer at an early stage [74]. Liquid biopsy plays an essential role in detecting early-stage lung cancer or minimal residual disease (MRD), aiding in treatment planning. Despite its high sensitivity and minimally invasive nature, it has challenges, including false positives, high costs, and reproducibility issues [75]. Post-surgical care prioritizes regular CT scans and potential adjuvant therapy to improve patient prognosis. Additionally, the low concentration of cfDNA in early-stage lung cancer can affect detection accuracy, making it a critical factor in the effectiveness of liquid biopsy. Liquid biopsy is a minimally invasive method for detecting cancer biomarkers in blood. Its strengths include high sensitivity, ease of integration with screening programs, and reduced patient discomfort [76]. However, it has limitations, such as false positives due to clonal hematopoiesis, high costs, and reproducibility challenges [77,78]. Despite these drawbacks, liquid biopsy remains a promising tool for early cancer detection and monitoring treatment responses. Multiomics approaches enhance the effectiveness of LDCT screening by integrating clinical, imaging, and biological data [79,80]. On one side, the benefits of this method include identifying high-risk non-smokers, optimizing sensitivity and specificity, reducing overdiagnosis, avoiding unnecessary treatments, and maintaining high-quality lung cancer screening. On the other side, the screening process is illustrated, featuring a human figure undergoing LDCT imaging and a liquid biopsy to assess lung cancer risk. The combination of LDCT screening and liquid biopsy enables precise risk stratification, improving early detection. A recent study developed a cost-effective shallow genome-wide cfDNA sequencing method that achieved high diagnostic accuracy (AUC 0.97) for lung cancer detection, demonstrating potential as a future alternative or complement to traditional LDCT screening due to its improved cost-efficiency and performance across all cancer stages [81]. By incorporating personalized screening strategies, this approach enhances accuracy and strengthens lung cancer prevention efforts.

(4) **Genomic Profiling and PRSs.**

The integration of PRS and genomic profiling into lung cancer screening presents several potential benefits [82]. First, it enables a more refined risk stratification beyond smoking history alone. This is particularly relevant in Asian populations, where a substantial proportion of lung cancer cases occur in non-smokers, often due to genetic predisposition and environmental exposures, such as air pollution or household fumes. Second, genomic-based models can improve the cost-effectiveness of screening by reducing unnecessary imaging and follow-up in low-risk individuals while prioritizing those at genuinely elevated risk. This aligns with the broader goal of precision public health—delivering the right intervention to the right person at the right time. However, several challenges remain. Wang et al. assessed lung cancer PRSs using two methods in 17,166 cases and 12,894 controls of European ancestry [42,83]. They found significant variability in individual-level PRS estimates, with modest correlations between methods. Incorporating confidence intervals improved risk prediction accuracy, highlighting the importance of considering individual-level uncertainty in clinical applications of the PRS [83]. The development and validation of population-specific PRS models are essential, as the performance of the PRS varies by ancestry. Most existing PRS models are derived from European-ancestry cohorts, limiting their generalizability to Asian or African populations [83]. Additionally, ethical considerations surrounding genetic risk communication, data privacy, and the psychological impact of knowing one’s genetic risk must be addressed. To fully leverage the potential of the PRS and genomic profiling in lung cancer screening, large-scale prospective studies are needed to validate their predictive utility across diverse populations. Integrating genomic data with clinical, imaging, and environmental information using machine learning approaches may further enhance predictive accuracy. Moreover, the implementation of SDM frameworks will be crucial to guide individuals through genetic risk-informed screening decisions. In summary, genomic profiling and the PRS hold transformative potential for lung cancer screening. By enabling more precise and equitable risk assessment, they represent a key step toward individualized, data-driven prevention strategies in oncology.

(5) **Government Policy and Human Behavioral and Environmental Data Integration**

In the implementation of cancer screening programs, government policy plays a pivotal role, not only in determining the efficiency of screening execution but also in significantly influencing public participation and uptake rates [84]. Although lung cancer screening has been shown to reduce mortality and facilitate stage shifts through early detection, it also carries potential drawbacks, such as overdiagnosis, overtreatment, false positives, and the unnecessary anxiety these may cause [3]. Therefore, alongside policy promotion, it is essential to integrate human behavioral data, environmental factors, and health literacy information [44,85]. Medical institutions should also offer SDM mechanisms to help individuals better understand the benefits and potential harms of lung cancer screening [86]. This approach is key to improving screening quality while minimizing unintended consequences.

Studies have indicated that performing lung cancer screening on populations that do not meet high-risk criteria can lead to an increased risk of overdiagnosis as the volume and coverage of screening expand—particularly among women [31,87]. This highlights the importance of risk-based screening policies, supported by personalized strategies and effective health communication, to maximize benefits while effectively managing associated risks [13,88].

Screening policies represent a vital component of preventive-oriented welfare strategies in public health, aiming to detect diseases at an early stage, reduce mortality rates, and alleviate the overall burden on healthcare systems [89]. However, the development and implementation of such policies must take into account the fairness and efficiency of resource allocation—particularly whether these resources are effectively targeted toward truly high-risk populations [90]. If screening efforts are broadly applied to low-risk individuals without precision, it may result in resource waste and an increased risk of overdiagnosis and overtreatment [87]. In addition, when healthcare institutions implement screening policies, an excessive emphasis on quantitative performance metrics—such as the number of examinations or KPI achievement rates—can lead to the neglect of screening quality, the completeness of follow-up care, and the actual needs of patients [87]. Screening should not be limited to the diagnostic procedure itself; it must be integrated with risk assessment, clinical judgment, psychological support, and personalized decision-making guidance. At the individual level, advances in minimally invasive thoracic surgery and shorter postoperative recovery periods have led many clinicians and patients to prefer relatively safe interventions, such as wedge resection. However, this trend may result in surgical intervention even for tumors with indolent behavior and without confirmed clinical malignancy, thereby increasing the risk of overtreatment and overdiagnosis [9]. Conversely, adopting an “active surveillance” strategy to monitor lesions can help avoid unnecessary immediate interventions [91]. Yet, from the patient’s perspective, prolonged periods of uncertainty and the psychological burden of “watchful waiting” may lead to anxiety, insomnia, excessive healthcare utilization, and a reduced quality of life, ultimately impacting their confidence in medical decision-making [92]. This long-term psychological impact is often underestimated in clinical practice but should be an essential consideration in screening policy design. Therefore, an effective and precise cancer screening policy must be grounded in the principles of risk stratification, quality-driven value-based care, and SDM [53]. Such a policy must also be supported by scientific evidence and intersectoral collaboration, ensuring that—from policy planning to clinical implementation—healthcare resources are used efficiently while protecting patient rights and mental well-being. Only through this comprehensive approach can we truly achieve the balance and mutual benefits of early detection, mortality reduction, and minimization of unnecessary medical interventions.

## 5. Implementing Precision Screening in Practice

Implementing precision screening in clinical practice represents a transformative shift from traditional population-based approaches toward a more individualized and data-driven model [93]. This transition requires comprehensive systems-level changes that encompass infrastructure, technology, clinical workflows, and ethical considerations. One of the foundational components is the establishment of a robust data infrastructure. This includes the integration and interoperability of electronic health records (EHRs), biobanks, and imaging repositories, which are essential for supporting multifactorial risk modeling [94]. By linking genomic, clinical, lifestyle, and imaging data, healthcare systems can more accurately stratify individuals based on their personalized risk for specific diseases, such as cancer or cardiovascular conditions [95]. The harmonization of these datasets enables longitudinal monitoring and supports predictive analytics that guide screening decisions. In parallel, clinical decision support tools (CDSTs), particularly those powered by AI, play a critical role in operationalizing precision screening. AI-driven dashboards can assist clinicians in identifying high-risk individuals, recommending optimal screening intervals, interpreting complex biomarkers, and managing incidental findings with greater accuracy and efficiency [96]. These tools not only enhance clinical workflow but also reduce variability in care delivery, ensuring more consistent application of screening protocols. However, the implementation of precision screening is not solely a technological endeavor—it necessitates meaningful patient engagement. SDM is a core principle, ensuring that patients understand their individualized risk, along with the potential benefits and harms of screening options. Empowering patients through risk communication tools, visual aids, and personalized consultations fosters informed choices and strengthens trust in medical recommendations [97]. Moreover, the success of precision screening depends on its equitable design and deployment. There is a pressing need to address potential disparities that may arise from algorithmic bias, access to genomic testing, or digital literacy gaps. Precision tools must be developed and validated using data from diverse populations to avoid exacerbating existing health inequities [98]. Additionally, culturally sensitive outreach strategies and inclusive implementation models are essential to ensure that underserved communities can benefit from advancements in personalized screening [99]. Ultimately, the integration of precision screening into routine care requires strategic planning, a professional workforce, interdisciplinary collaboration, and policy support. Investments in health IT infrastructure, clinician training, ethical governance, and patient education are all pivotal. When executed thoughtfully, precision screening has the potential to enhance early detection, reduce overdiagnosis, and optimize health outcomes by aligning screening strategies with the unique characteristics of each individual.

The successful implementation of precision screening relies not only on advanced technologies and data integration but also on the capacity and well-being of the healthcare workforce, including radiologists, radiographers, thoracic surgeons, and clinical physicians [100]. As these professionals face increasing demands—from interpreting complex multimodal data to engaging in SDM and multidisciplinary coordination—the risk of burnout becomes a critical issue. Radiologists, in particular, are experiencing heightened cognitive load due to growing imaging volumes and the need for precise interpretation in the context of personalized risk models. Radiographers and physicians are also navigating evolving roles as they support patient-centered communication and manage incidental findings. To alleviate this pressure, AI and CDSTs can offer valuable assistance by automating routine tasks, flagging high-risk cases, and streamlining workflows [49]. These technologies should be designed to complement, not replace, clinical expertise—enabling healthcare providers to focus on complex decision-making and patient care. Ensuring a sustainable precision screening ecosystem requires workforce support strategies, such as training, workload balancing, interdisciplinary collaboration, and organizational policies that prioritize clinician well-being.

The transition from traditional cancer screening to precision screening necessitates a systems-level transformation and reform. First, in terms of data infrastructure, the integration and interoperability of EHRs, biobanks, and imaging repositories are fundamental to enabling multifactorial risk modeling [101]. This facilitates real-time data sharing across care teams and supports clinical decision-making. Second, the implementation of CDSTs—particularly AI-enabled dashboards—can assist clinicians in identifying high-risk individuals, recommending personalized screening intervals, and efficiently managing incidental findings, thereby improving decision-making accuracy and efficiency.

Patient engagement is also a key element in the success of precision screening. Through SDM, patients should be informed about their individual risk, along with the potential benefits and harms of screening, enabling them to make health decisions that align with their personal values [102,103]. Meanwhile, equity considerations must not be overlooked. Screening systems and algorithms should be designed to prevent exacerbating existing health disparities. Training data must be diverse, and access to services must be equitably distributed. In addition, the implementation of precision screening faces an urgent need for healthcare workforce development [104]. There is a pressing demand to train professionals with expertise in genomics, AI applications, health data interpretation, and culturally sensitive communication to meet the complex challenges of future precision medicine. Looking ahead, the development of precision lung cancer screening is closely aligned with broader trends in digital health, genomic medicine, and personalized care, and it is poised to reshape screening strategies and practices. First, integrated screening platforms are expected to become standard. The combination of LDCT, liquid biopsy, and AI-based image analysis will significantly enhance diagnostic sensitivity and specificity. Second, dynamic risk models will continuously update individual risk profiles in real-time based on evolving health data, imaging results, and lifestyle changes, resulting in more accurate predictions [105]. Regarding personalized screening intervals, the “one-size-fits-all” annual scan approach will be replaced. Screening frequency will be tailored based on individual risk evolution, with some individuals requiring more frequent screening and others less [106]. Finally, promoting globally and population-specific strategies is crucial. Culturally and contextually tailored approaches for populations, such as non-smoking Asians, women, and residents of rural areas, will help expand the real-world impact of screening programs and ensure truly equitable and accessible precision screening [13].

## 6. A New Paradigm in Early Detection: 4P-Oriented Precision Lung Cancer Screening

**Figure 2:**
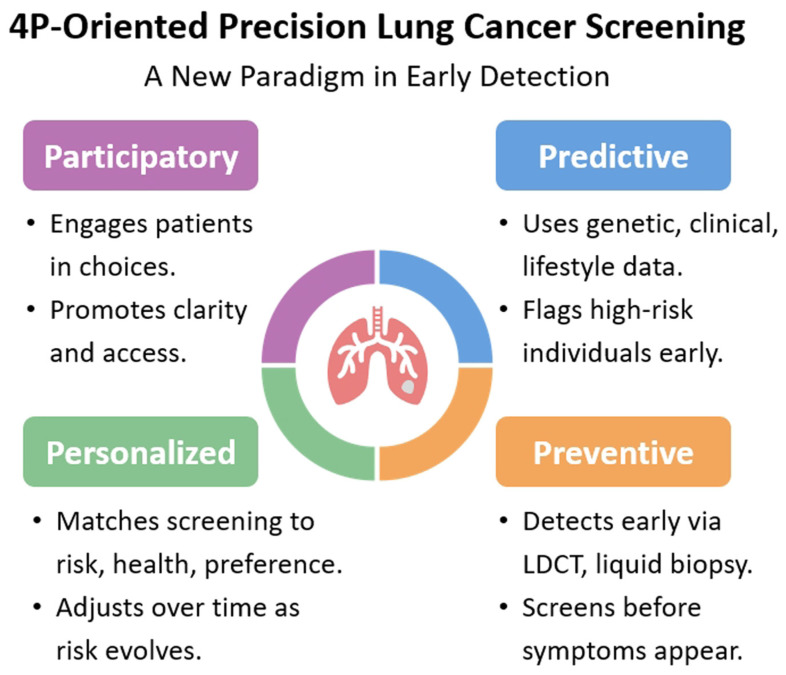
**The 4P-oriented precision lung cancer screening framework.** The 4P approach to early lung cancer detection—participatory, predictive, preventive, and personalized. Each domain emphasizes a core component of patient-centered precision screening. Participatory screening promotes shared decision-making and access; predictive approaches use genetic, clinical, and lifestyle data to identify high-risk individuals early; preventive strategies focus on early detection before symptoms arise using tools like LDCT and liquid biopsy; and personalized screening tailors interventions to individual risk, health status, and preferences, adjusting as risks evolve.

The future of lung cancer screening is shifting toward a 4P model—predictive, preventive, personalized, and participatory—to enhance early detection, reduce harm, and ensure equitable outcomes, as shown in Figure 2 [107]. Predictive approaches leverage multifactorial risk models that integrate genetic, clinical, environmental, and lifestyle data to identify individuals at highest risk before symptoms emerge. Preventive strategies shift the focus from treatment to early intervention through timely screening of high-risk groups using tools, like LDCT, and emerging biomarkers, such as liquid biopsy [108]. Personalized screening tailors intervals, modalities, and follow-up strategies based on each individual’s evolving risk profile, comorbidities, and preferences. Participatory elements empower individuals through SDM, clear communication of risks and benefits, and inclusive program design that ensures access across diverse populations. This 4P approach reflects a systems-level transformation in lung cancer control—combining advanced data analytics, digital health innovations, and patient-centered care to deliver smarter, safer, and more effective screening programs.

**Figure 3:**
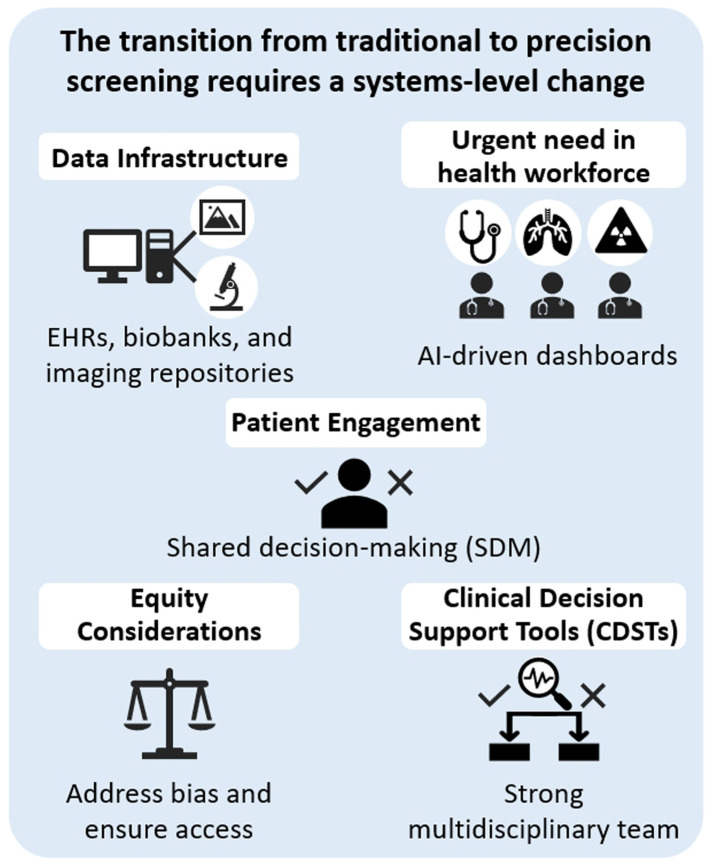
**Systems-level requirements for transitioning to precision screening.** Key components include robust data infrastructure (e.g., EHRs, biobanks, imaging), a trained health workforce using AI-enabled tools, and enhanced patient engagement through shared decision-making (check and cross indicate decisions in SDM process). Equity considerations ensure that access and bias are addressed. The implementation of clinical decision support tools (CDSTs) by multidisciplinary teams is critical for accurate, personalized decision-making.

The transition from traditional to precision screening requires a systems-level change, as shown in Figure 3, including the following:

(1) **Data Infrastructure**: EHRs, biobanks, and imaging repositories must be integrated and interoperable to support multifactorial risk modeling [109].
(2) **CDSTs**: AI-driven dashboards can aid clinicians in identifying high-risk patients, recommending screening intervals, and managing incidental findings [86].
(3) **Patient Engagement**: While lung cancer screening uptake rates remain relatively low in the United States, they tend to be higher in several Asian populations. This disparity may be attributed to differences in cultural norms, health literacy levels, and cancer-related perceptions, highlighting the need for culturally tailored patient engagement strategies [102,110,111,112,113]. SDM is crucial in precision screening. Patients should be educated about their personalized risk, potential benefits, and harms of screening to make informed choices.
(4) **Equity Considerations**: Screening programs must ensure that precision tools do not inadvertently widen disparities. For example, algorithms should be trained on diverse populations to avoid bias and ensure equitable access [98].
(5) **Urgent Need in the** Health Workforce: There is an urgent need to strengthen the health workforce for lung cancer screening. A multidisciplinary team—including radiologists, clinical physicians, and thoracic surgeons—is essential to ensure accurate diagnosis, timely treatment, and appropriate follow-up, especially as screening programs expand and early-stage lung cancers are increasingly detected in asymptomatic individuals [100]. In addition, nursing educators involved in fast-track screening pathways should also possess adequate health literacy on screening. An integrated, streamlined approach is essential to optimize both the overall screening process and health literacy education.

## 7. The Future of Precision Lung Cancer Screening

**Figure 4:**
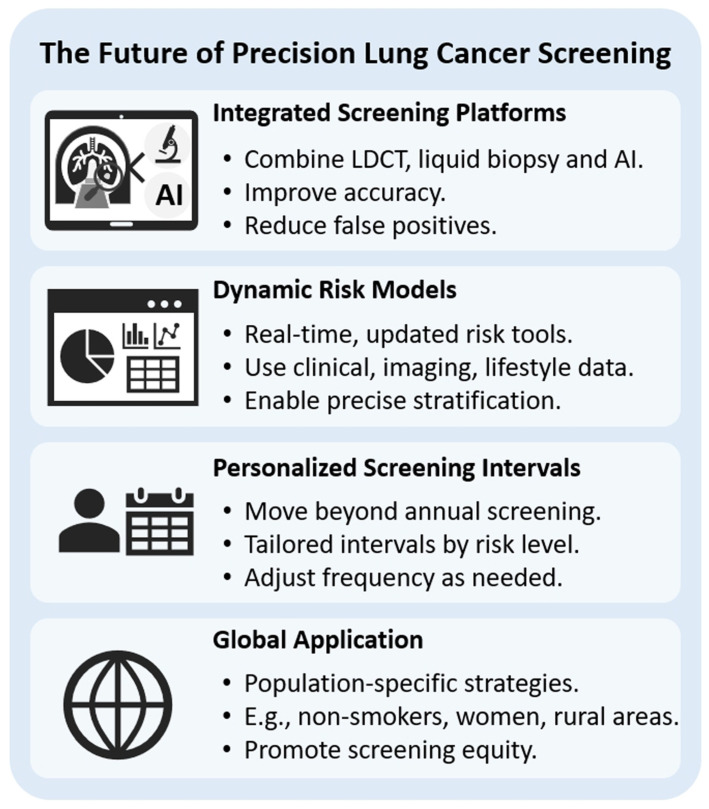
**The future of precision lung cancer screening.** Integrated screening platforms that combine LDCT, liquid biopsy, and AI aim to improve accuracy and reduce false positives. Dynamic risk models use real-time, multi-source data for precise risk stratification. Screening intervals are becoming more personalized based on individual risk profiles. Lastly, global application calls for population-specific strategies to address disparities and ensure equity in screening access, especially for underrepresented groups, such as non-smokers, women, and those in rural areas.

The evolution of precision medicine in lung cancer screening is aligned with broader trends in digital health, genomics, and personalized care. In the near future, we can expect several key developments, as shown in Figure 4. (1) **Integrated Screening Platforms**—Screening strategies will increasingly combine LDCT with liquid biopsy and AI-powered image analysis to enhance diagnostic accuracy and reduce false positives. (2) **Dynamic Risk Models**—Risk assessment tools will shift from static evaluations to real-time, continuously updated models that incorporate new clinical data, imaging findings, and lifestyle changes, allowing for more precise stratification. (3) **Personalized Screening Intervals**—Instead of uniform annual screenings, patients will be assigned tailored intervals based on their individual risk trajectories, with some requiring more frequent scans, while others may need them less often [114,115].(4) **Global Application**—There will be greater emphasis on developing population-specific screening strategies, such as for non-smoking Asians, women, or residents of rural areas, to ensure that the benefits of lung cancer screening are equitably distributed and optimized across diverse demographic groups [116,117].

## 8. Conclusions

Precision medicine offers a promising path forward in lung cancer screening, moving beyond static, smoking-based criteria to a holistic, individualized approach. By integrating genomics, advanced imaging, biomarkers, and AI, precision screening can more effectively identify those at greatest risk, reduce unnecessary interventions, and ultimately save more lives. As technologies evolve and evidence accumulates, precision screening will likely become the new standard of care, offering a more intelligent and humane approach to lung cancer prevention.

## Figures and Tables

**Table 1:** Four screening eligibility guidelines and performance criteria.

Metric	CGSL	NCCN	USPSTF	I-ELCAP
Eligibility rate ^1^	13.92%	6.97%	6.81%	53.46%
Efficiency ratio (ER) ^2^	1.46%	1.64%	1.51%	1.13%
Inclusion rate ^3^	19.0%	9.5%	9.3%	73.0%
Proportion of detected lung cancers ^4^	29.2%	16.4%	14.8%	86.6%

Definitions: ^1^ Eligibility rate: Percentage of individuals meeting the guideline’s criteria for screening. ^2^ Efficiency ratio (ER): Proportion of diagnosed lung cancer cases among those eligible for screening. ^3^ Inclusion rate: Percentage of total lung cancer cases detected among those eligible for screening. ^4^ Proportion of detected lung cancers: Percentage of total diagnosed lung cancer cases that met the guideline’s screening criteria.

## Data Availability

The raw data supporting the conclusions of this article will be made available by the authors on request.

## Abbreviations

LDCT	low-dose computed tomography
SSNs	subsolid nodules
USPSTF	United States Preventive Services Task Force
ER	efficiency ratio
LCS	lung cancer screening
CGSL	China guideline for the screening and early detection of lung cancer
NCCN	National Comprehensive Cancer Network
I-ELCAP	International Early Lung Cancer Action Program
AI	artificial intelligence
PRSs	polygenic risk scores
SNPs	single-nucleotide polymorphisms
MCED	multi-cancer early detection
SDM	shared decision-making
GGNs	ground-glass nodules
IPA	invasive pulmonary adenocarcinoma
S-RRL	serial longitudinal radiomics-based RRL model
CEA	carcinoembryonic antigen
ctDNA	circulating tumor DNA
MRD	minimal residual disease
EHRs	electronic health records
CDSTs	clinical decision support tools
